# Breast cancer risk factors in Iranian women: a systematic review and meta-analysis of matched case–control studies

**DOI:** 10.1186/s40001-022-00952-0

**Published:** 2022-12-27

**Authors:** Malihe Khoramdad, Masoud Solaymani-Dodaran, Ali Kabir, Neda Ghahremanzadeh, Esmat-o-Sadat Hashemi, Noushin Fahimfar, Zahra Omidi, Mohammad Ali Mansournia, Asiie Olfatbakh, Hamid Salehiniya, Shahpar Haghighat

**Affiliations:** 1 Department of Epidemiology, School of Public Health, Iran University of Medical Sciences, Tehran, Iran; 2 Minimally Invasive Surgery Research Center, Hazrat-E-Rasool Hospital, Iran University of Medical Science, Tehran, Iran; 3 Breast Cancer Research Center, Motamed Cancer Institute, ACECR, Tehran, Iran; 4 Osteoporosis Research Center, Endocrinology and Metabolism Clinical Sciences Institute, Tehran University of Medical Sciences, Tehran, Iran; 5 Department of Epidemiology and Biostatistics, School of Public Health, Tehran University of Medical Sciences, Tehran, Iran; 6 Social Determinants of Health Research Center, Birjand University of Medical Sciences, Birjand, Iran

**Keywords:** Risk factors, Breast neoplasms, Meta-analysis, Case–control studies

## Abstract

**Background:**

Identifying breast cancer risk factors is a critical component of preventative strategies for this disease. This study aims to identify modifiable and non-modifiable risk factors of breast cancer in Iranian women.

**Methods:**

We used international databases (PubMed/Medline, Scopus, Web of Knowledge, and Embase) and national databases (SID, Magiran, and ISC) to retrieve relevant studies until November 13, 2022. The odds ratio (OR) with a 95% confidence interval using the random-effect model was used to estimate the pooled effect. The publication bias was assessed by the Egger and Begg test. A sensitivity analysis was conducted to evaluate the effect of each included study on the final measurement.

**Results:**

Of the 30,351 retrieved articles, 24 matched case–control records were included with 12,460 participants (5675 newly diagnosed cases of breast cancer and 6785 control). This meta-analysis showed that of the known modifiable risk factors for breast cancer, obesity (vs normal weight) had the highest risk (OR = 2.17, 95% CI 1.47 to 3.21; *I*^2^ = 85.7) followed by age at marriage (25–29 vs < 18 years old) (OR = 2.00, 95% CI 1.53 to 2.61; *I*^2^ = 0), second-hand smoking (OR = 1.86, 95% CI 1.58 to 2.19; *I*^2^ = 0), smoking (OR = 1.83, 95% CI 1.41 to 2.38; *I*^2^ = 18.9), abortion history (OR = 1.44, 95% CI 1.02 to 2.05; *I*^2^ = 66.3), oral contraceptive use (OR = 1.35, 95% CI 1.11 to 1.63; *I*^2^ = 74.1), age at marriage (18–24 vs < 18 years old) (OR: 1.22, 95% CI 1.02 to 1.47; *I*^2^ = 0). Of non-modifiable risk factors, history of radiation exposure (OR = 3.48, 95% CI 2.17 to 5.59; *I*^2^ = 0), family history of breast cancer (OR = 2.47, 95% CI 1.83 to 3.33; *I*^2^ = 73), and age at menarche (12–13 vs ≥ 14 years old) (OR = 1.67, 95% CI 1.31–2.13; *I*^2^ = 25.4) significantly increased the risk of breast cancer.

**Conclusions:**

Since most risk factors related to breast cancer incidence are modifiable, promoting healthy lifestyles can play an influential role in preventing breast cancer. In women with younger menarche age, a family history of breast cancer, or a history of radiation exposure, screening at short intervals is recommended.

**Supplementary Information:**

The online version contains supplementary material available at 10.1186/s40001-022-00952-0.

## Introduction

Women's breast cancer is the leading cause of cancer incidence in 2020, with about 2.3 million new cases, accounting for 11.7% of all cancer cases (1 in 4 cancer cases). It is the fifth leading cause of cancer mortality worldwide, with 685,000 deaths (1 in 6 cancer deaths) [1]. In Iran, breast cancer is the most frequent cancer among females [2]. Not only is the incidence increasing [3, 4], but also people with the disease are on average ten years younger than their Western counterparts [5]. It has been introduced leading cause of cancer mortality in women (age-standardized rate = 10.8 per 100,000) [6].

Breast cancer risk factors are divided into modifiable or lifestyle risk factors, which can be prevented, and non-modifiable risk factors [7]. Identifying these risk factors plays a significant role in primordial, primary, and secondary prevention. Breast cancer incidence varies widely among different populations globally [1]. So, it seems that there are no similar risk factors for all countries, and each country must identify the risk factors based on its demographic characteristics.

To identify risk factors, cohort studies are the best type of study, but case–control studies can also be an excellent alternative choice when the disease of interest is rare [8]. In Iran, there is no prospective study about breast cancer, and many conducted studies to identify risk factors of breast cancer are case–control studies, but their results vary. So, studies should be pooled to achieve consensus.

In this regard, a meta-analysis study was conducted in 2020 [9], but this study had some methodological shortcomings, such as a lack of comprehensive search strategy, combining of studies with different designs (case–control and cross-sectional studies), pooling of incident or prevalent cases in case–control studies, unclassified matched and unmatched case–control studies. These methodological problems can increase the recall and information bias and prevalent cases are mostly a sample of long disease duration and survival, so cannot be representative of the general population status. Besides the combination of studies with adjusted and crude odds ratios and including studies with different or non-defined reference categories in pooled estimation were important limitations of the published meta-analysis study which need to be considered in the current research.

Considering mentioned limitations of using prevalent cases, no access to cohort studies in Iran, and more efficient matched case–control studies than unmatched ones [10], we aimed to conduct a systematic review and meta-analysis study of matched case–control studies to determine breast cancer risk factors in Iranian women.

## Materials and methods

This systematic review and meta-analysis were carried out using the Meta-analysis Of Observational Studies in Epidemiology (MOOSE) guideline [11]. A protocol was not registered at the international prospective register of systematic reviews (PROSPERO).

### Search strategy

We used PubMed/Medline, Scopus, Web of Knowledge, Embase, and the Iranian database (SID, Magiran, and ISC) to retrieve the observational studies on breast cancer risk factors in Iran until November 13, 2022. Two groups of keywords were used for defining the breast cancer risk factors: the most important breast cancer risk factors presented in research worldwide and compatible with the MeSH library, and the keywords with the meaning of association such as correlation, relationship, etc. To find additional related studies, references of included studies, conducted systematic reviews, and meta-analyses were used. The search strategy details are presented in Additional file 1: Appendix S1 (Table A–D).

### Eligibility criteria

The PECOS statement (Population, Exposure, Comparison, Outcomes, and Study design) is a framework to formulate eligibility criteria in systematic reviews. The research question was conducted using the PECOS framework (Table 1).

**Table 1:** Research question based on the PECOS framework

Population	Iranian women
Exposure	Females with specific risk factors
Comparisons	Females without specific risk factors
Outcome	Incidence of malignant breast cancer in the last year with pathology confirmation
Study design	Matched case–control

To be included in the meta-analysis, a published study had to meet the following criteria: (1) being original article, (2) published in Persian or English language, (3) compliance with PECOS criteria. Exclusion criteria consisted of the control group selection from patients with benign breast disease, matching on numerous variables, matching on variables except for age. Not reporting the odds ratio (OR) or not being able to calculate OR and the 95% confidence interval.

To find additional studies, we used the reference of the included studies and the systematic reviews and meta-analyses studies. If there were several publications from a dataset, the article presenting the most risk factors was selected. If there were different risk factors in those publications, all of them were included in the study.

### Data extraction

The search results of all databases were combined using EndNote, and duplicates were deleted. Two researchers (MKH and NGH) who were blinded to authors and journal names, reviewed the publications to identify those meeting the eligibility criteria being. A third author (SHH) addressed the possible lack of consensus between the two authors. There was 95% inter-author reliability by kappa statistics.

If the full text was not accessible or the type of selection cases (incident/prevalent cases) or controls were ambiguous, the corresponding authors were contacted by email for further data. After selecting the final records, two authors (MKH and NGH) started data extraction. Data included titles, first author's name, study design, sample size, publication year, patient recruitment period, city, study setting, risk factors, case and control description, number of cases and controls, number of exposed cases with risk factors, and number of exposed controls with risk factors, matching factors, crude and adjusted OR with 95% confidence interval (CI).

### Association measurement

The measure of association between exposure and occurrence of disease in case–control studies is the odds ratio (OR). If there was no OR in a study, we calculated OR from the data of the article. OR refers to the odds of exposure to a specific risk factor in women with breast cancer compared to control group. Selected controls were matched on the various variables and age was common in all of them. A list of matched variables is reported in Table 2.

**Table 2:** Characteristics of included studies

First author year	City	Patient recruitment period	Sample size (case, control)	Mean age	Study setting	Selection of controls	Description of case group	Description of control group	Matching factors	Risk factors^a^
Maleki 2020 [24]	Tehran	September 2011 to May 2016	1925 (958, 967)	Case: 46.0 ± 10.1 Control: 46.1 ± 10.2	Imam Khomeini Hospital	Hospital-based	▪ Patients admitted for treatment of breast cancer in surgical and chemotherapy wards ▪ Histopathological confirmation of new breast cancer within the past 12 months ▪ At least 18 years of age ▪ No history of concurrent cancer in other organs	Healthy relatives or friends of hospitalized patients for diseases other than cancer at the same hospital and at the same time	▪ Age (± 5) ▪ City of residence	Age, occupation, education, physical activity, BMI, parity, age at first pregnancy, OCP use, duration of OCP use, breastfeeding history, breastfeeding duration, age at menarche, family history of breast cancer
Safabakhsh 2020 [48]	Tehran	September 2017 to June 2018	300 (150, 150)	Case: 46.6 ± 10.7 Control: 46.1 ± 10.7	Imam Khomeini Hospital	Hospital-based	▪ Women with breast cancer ▪ Histopathological confirmation of new breast cancer within the past three months ▪ 24–73 years of age ▪ No history of breast or other cancers	▪ Healthy relatives of hospitalized patients at the same hospital in the other wards such as dermatology, urology, orthopedic, etc. ▪No family relationship with cases	Age	Age, education, marital status, SES, height, weight, BMI, number of live children, breastfeeding duration, duration of OCP use, HRT, physical activity, smoking, alcohol use, supplement intake, dietary pattern, menopause status, age at menopause, age at menarche, family history of breast cancer
Sasanfar 2019 [26]	Tehran	2014 to 2016	868 (412, 456)	Case: 46.3 ± 10 Control: 44.2 ± 11.3	Imam Khomeini Hospital	Hospital-based	▪ Breast cancer patients referred to surgery, chemotherapy, or radiotherapy department ▪ Histopathological confirmation of new breast cancer within the past 12 months ▪ No history of any other cancers No long-term dietary restrictions ▪ No total energy intake of > 4500 or < 800 kcal ▪ No response to more than 70 items of FFQ	▪ Healthy relatives or friends of hospitalized patients for diseases other than cancer at the same hospital ▪ No long-term dietary restrictions ▪ No total energy intake of > 4500 or < 800 kcal ▪ No response to more than 70 items of FFQ	▪ Age (± 10 years) ▪ City of residence	Age, education, marital status, physical activity, BMI, dietary pattern, smoking, alcohol use, OCP use, HRT, infertility treatment, parity, age at menarche, family history of breast cancer
Vahid 2018 [19]	Tehran	March 2015 to February 2016	293 (145, 148)	Case: 49.83 ± 11.86 Control: 48.54 ± 12.0	Cancer Research Center (Shahid Beheshti University of Medical Sciences)	Hospital-based	▪ Patients with breast cancer who were diagnosed by a pathologist within the previous month ▪ Selection of eligible patients randomly	Hospitalized female patients in the same center randomly	Age (± 10)	Age, occupation, education, marital status, BMI, smoking, physical activity, dietary pattern, parity, breastfeeding history, OCP use, HRT, menopause status, age at menarche, family history of breast cancer
Heidari 2018 [49]	Tehran	September 2015 to February 2016	401 (134, 267)	Case: 49.49 ± 10.68 Control: 47.13 ± 10.08	▪ Imam Hossein Hospital ▪ Shohada Hospital	Hospital-based	▪ Breast cancer patients admitted to the two referral hospitals in Tehran city ▪ Histopathological confirmation of new breast cancer within the past six months	Hospitalized female patients in other sections of the same hospitals for a broad spectrum of non-neoplastic diseases that were unrelated to alcohol abuse, smoking, and long-term diet modification	▪ Age(± 5) ▪ Menopause status	Age, education, height, weight, BMI, WHR, breastfeeding duration, physical activity, age at marriage, age at first live birth, OCP use, smoking, supplement intake, dietary pattern, day bra use, night bra use, age at menarche, menopause status, family history of cancer
Fararouei 2018 [22]	Shiraz	November 2014 to March 2016	1010 (505, 505)	Case: 41.78 ± 10.56 Control: 42.24 ± 10.62	Namazi Hospital	Hospital-based	▪ Patients admitted for treatment of breast cancer in surgical and radiotherapy wards ▪ Histopathological confirmation of new breast cancer ▪ 20–55 years of age ▪ Without any recent significant change in their weight or size from the last six months	Hospitalized female patients for diseases other than cancer at the same hospital randomly	Age (± 5)	Age, education, occupation, marital status, age at marriage, BMI, physical activity, OCP use, smoking, second-hand smoking, dietary pattern, family history of breast cancer
Dianatinasab 2017 [21]	Shiraz	November 2014 to March 2016	1052 (526, 526)	Case: 47.8 ± 10.58 Control: 46.75 ± 11.08	Namazi Hospital	Hospital-based	▪ Patients admitted to the hospital due to breast cancer ▪ Histopathological confirmation of new breast cancer ▪ Without a change in their weight in the past 6 months	Hospitalized female patients in the same hospital without having any hormonal, gynecology, cancer diseases, or change in their weight in the past 6 months	Age	Age, occupation, education, marital status, place of residence, age at marriage, BMI, physical activity, smoking, second-hand smoking, hair coloring, cosmetic use, day sleep duration, quality sleep, regular bedtime, past life stress, menopause status, age at first delivery, parity number, birth interval, prenatal age, abortion history, birth weight, OCP use, breastfeeding duration, chest X-ray history, hysterectomy, age at menarche, family history of breast cancer
Pourzand 2016 [50]	Tabriz	January 2012 to June 2013	582 (285, 297)	Case: 46.4 ± 10.02 Control: 41.4 ± 9.6	Shams Hospital	Hospital-based	▪ Women with breast cancer admitted to the surgery ward of the hospital ▪ Newly diagnosed breast cancer patient ▪ age of 25 to 65 years ▪ No prior history of histologically confirmed malignancies ▪ No previous history of cancer ▪ No history of cystic abnormalities or benign breast disease ▪ Not a pregnant, postpartum, or breastfeeding mother ▪ Not following a specific dietary habit ▪ Not having disorders of polycystic ovary syndrome and chronic inflammatory disorders ▪ No chronic use of methotrexate, sulfasalazine, anticonvulsants, and contraceptive drugs ▪ Body mass index (BMI) < 45 kg/m2	▪ Hospitalized female patients at the same hospital for no neoplastic disease ▪ No prior history of malignancies and benign neoplasms ▪ The will to participate in the study ▪ Not pregnant, postpartum, and breastfeeding mother ▪ Not following a vegetarian diet ▪ Not having chronic inflammatory disorders	▪ Age at diagnosis (± 5) ▪ Residence status (rural/urban and/or province)	Age, occupation, BMI, physical activity, menopause status, pregnancy number, number of lactation, dietary pattern
Montazeri 2016 [51] [In Persian]	Tehran Tabriz	2007–2014	975 (432, 543)	Case: 48.6 ± 4.7 Control: 40.6 ± 10.7	▪ Shohadaye Tajrish Hospital ▪ Taleghani Hospital Pars Hospital ▪ Azar Clinic ▪ Khatam Clinic ▪ Day Clinic ▪ Ghazi Hospital ▪ Imam Reza Hospital ▪ Noor Nejat hospital	Hospital-based	▪ Women with admitted breast cancer to the surgery ward of Shohadaye Tajrish, Imam Reza, Noor Nejat Hospitals, Azar, Khatam, and Day Clinics, Also women with admitted breast cancer to the radiotherapy and chemotherapy ward of Shohadaye Tajrish, Pars, Taleghani, and Ghazi Hospitals ▪ Histopathological confirmation of new breast cancer ▪ The will to participate in the study ▪ No previous history of cancer ▪ No prior history of benign breast disease ▪ No change in dietary pattern in the last five years ▪ No history of hormonal and gynecological diseases ▪ No chronic use of methotrexate, cyclosporine, metformin, aspirin, anticonvulsant and contraceptive drugs ▪ Not having autoimmune diseases ▪ No pregnancy or breastfeeding	▪ Healthy relatives of patients at the same hospital in the other wards such as dermatology and cosmetic surgery, urology, Ear noses and throat, gastroenterology orthopedic ▪ No previous history of malignant and benign mass ▪ No change in dietary pattern in the last five years ▪ No chronic use of methotrexate, cyclosporine, metformin, aspirin, anticonvulsant and contraceptive drugs ▪ No history of hormonal and gynecological diseases ▪ No pregnancy or breastfeeding	▪ Age (± 5) ▪ Place of residence	Age, age at first delivery, pregnancy number, breastfeeding duration, number of lactation, age at menarche, age at menopause
Salarabadi 2015 [52]	Kermanshah	2012 to 2014	151 (47, 105)	Case: 38.82 ± 5.37 Control: 38.89 ± 6.41	Imam Reza Hospital	Unknown	▪ Women with breast cancer admitted to the surgery ward of the hospital ▪ Histopathological confirmation of new breast cancer ▪ Without any menopausal evidence ▪ No pregnancy or breastfeeding ▪ No history of malignancy ▪ No familial history of breast cancer ▪ Were not exposed to PAHs producing manufacturers as well as X-ray resources according to their living and working addresses	▪ Normal healthy women ▪ Without any menopausal evidence ▪ No pregnancy or breastfeeding ▪ No history of malignancy ▪ No familial history of breast cancer ▪ Didn’t exposed to PAHs producing manufacturers as well as X-ray resources according to their living and working addresses	Age (± 5) SES	Age, sunlight exposure, physical activity, OCP use, dietary pattern, supplement intake
Arbabi 2014 [53]	Sabzevar	2010 to 2012	176 (60, 116)	Case: 36.45 ± 7.02 Control: 34.2 ± 5.7	Emdad Hospital	Unknown	▪ Women with breast cancer admitted to the surgery ward of the hospital ▪ Histopathological confirmation of new breast cancer ▪ Without any menopausal evidence ▪ No history of malignancy ▪ No pregnancy or breastfeeding ▪Dind't exposed to PAHs producing manufacturers as well as X-ray resources according to their living and working addresses	▪ Normal women ▪ Without any menopausal evidence ▪ No history of malignancy ▪ No pregnancy or breastfeeding ▪ Dind't exposed to PAHs producing manufacturers as well as X-ray resources according to their living and working addresses	Age (± 5)	Age, marital status, age at marriage, weight, height, BMI, age at first pregnancy, dietary pattern, supplement intake, age at menarche
Hosseinzadeh 2014 [54]	Tabriz	December 2012 to September 2013	448 (157, 291)	Case: 47.6 ± 10.7 Control: 46.8 ± 10.4	Imam Reza Hospital	Hospital-based	▪ Women with breast cancer admitted to the oncology ward of the hospital ▪ Histopathological confirmation of new breast cancer within the past 12 months	Female patients admitted in the same hospital in orthopedic, surgery, ear, nose, and throat, and trauma clinics without any neoplastic or hormonal disease and hysterectomy history	Age	Age, occupation, education, place of residence, marital status, income, age at marriage, smoking, second-hand smoking, hookah, drug user, alcohol use, exercise, stress, number of live births, abortion history, breastfeeding history, OCP use, HRT, age at menopause, menopause status, biopsy history, infertility therapy, diet containing sufficient fruit and vegetables, high-fat diet, migration, income
Sheikhi 2014 [25]	Tehran	April to May 2009	93 (53, 40)	Case: 40..2 ± 10.01 Control: 39.78 ± 11.21	Iranian Center for Breast Cancer, Academic Center for Education, Culture, and Research (ACECR)	Unknown	▪ Histopathological confirmation of new breast cancer within the past month ▪ Aged 20–65 years ▪ Residing in Tehran ▪ Without any other diagnosed diseases ▪ BMI ≤ 40 kg/m2 ▪ Without any type of special diet in the past two months ▪ No pregnancy or breastfeeding	▪ Healthy women ▪ Without any cancer ▪ Age 20–65 years ▪ Residing in Tehran ▪ Without any other diagnosed diseases ▪ BMI ≤ 40 kg/m2 Without any type of special diet in the past 2 months ▪ No pregnancy or breastfeeding	Unknown	Age, marital status, dietary pattern, WC, family history of breast disease or cancer, HRT, menopause status
Bahadoran 2013 [55]	Tehran	April 2010 to July 2010	274 (100, 174)	Case: 46..2 ± 9.3 Control: 45.9 ± 9.4	Shohada-e-Tajrish Hospital	Hospital-based	▪ Women with breast cancer admitted to the oncology, radiotherapy, chemotherapy, or surgery ward of the hospital ▪ Histopathological confirmation of new breast cancer within the past 5 months ▪ Aged 30–65 years ▪ Without any type of special diet ▪ No history of any cancer or cyst (excluding current breast cancer) ▪ No history of hormone therapy	▪ Women referring to outpatient clinics of the hospital, including gynecology and dermatology ▪ Without any type of special diet ▪ Without any history of cancers or cysts ▪ Without any acute or chronic diseases affecting the nutritional status ▪ Without any hormone therapy were recruited from among individuals	Age (± 5)	Age, occupation, physical activity, weight, BMI, age at menarche, familial history of breast cancer, smoking, diabetes, menopause status
Hajian 2013 [56] [In Persian]	Babol	April 2008 to September 2009	300 (100, 200)	Case: 51.2 ± 9.6 Control: 51.1 ± 9.3	▪ Beheshti Hospital ▪ Yahyanejad Hospital ▪ Hariri Cancer Screening Center	Hospital-based	▪ ▪ Patients in pathologic centers of two major educational hospitals and the Hariri cancer screening center ▪ ▪ Histopathological confirmation of new breast cancer	▪ Outpatient clinics in the same hospitals without any cancers and systemic diseases such as diabetes, hypertension, and cardiovascular disease ▪ Relatives of hospitalized patients without any cancers and systemic diseases such as diabetes, hypertension, and cardiovascular disease	Age (± 3)	Age, education, age at first pregnancy, age at menarche, marital status, place of residence, menopause status, exercise, familial history of cancer, history of radiation exposure
Hajian 2012 [57]	Babol	April 2008 to September 2009	300 (100, 200)	Case: 51.2 ± 9.6 Control: 51.1 ± 9.3	▪ Beheshti Hospital ▪ Yahyanejad Hospital ▪ Hariri Cancer Screening Center	Hospital-based	Histopathological confirmation of new breast cancer	▪ Outpatient clinics of the same hospitals without any cancers and systemic diseases such as diabetes, hypertension, and cardiovascular disease ▪ Relatives of hospitalized patients without any cancers and systemic diseases such as diabetes, hypertension, and cardiovascular disease	Age (± 3)	Age, education, age at first pregnancy, age at menarche, parity, abortion history, OCP use, smoking, exercise, BMI, menopause status, breastfeeding duration
Hajian 2011 [58]	Babol	April 2008 to September 2009	300 (100, 200)	Case: 51.2 ± 9.6 Control: 51.1 ± 9.3	▪ Beheshti Hospital ▪ Yahyanejad Hospital ▪ Hariri Cancer Screening Center	Hospital-based	Histopathological confirmation of new breast cancer	▪ Outpatient clinics in the same hospitals without any cancers and systemic diseases such as diabetes, hypertension, and cardiovascular disease ▪ Relatives of hospitalized patients without any cancers and systemic diseases such as diabetes, hypertension, and cardiovascular disease	Age (± 3)	Age at menarche, age at first pregnancy, number of pregnancies, number of live births, menopause status, breastfeeding duration, OCP use, abortion history
Rezaeiian 2012 [59] [In Persian]	Tehran	2007–2010	346 (177, 169)	Case: 49.1 ± 8.9 Control: 40.09 ± 11.4	Hospitals affiliated with Shahid Beheshti University of Medical Sciences	Hospital-based	▪ Patients admitted for treatment of breast cancer oncology ward ▪ Histopathological confirmation of new breast cancer ▪ Aged 30–60 years ▪ The will to participate in the study ▪ No history of any benign mass breast ▪ No change in dietary pattern in the last 3 years ▪ No history of hormonal and gynecological diseases ▪ No chronic use of methotrexate, cyclosporine, metformin, aspirin, anticonvulsant and contraceptive drugs	▪ Healthy women in the same hospital ▪ No history of any malignant or benign mass ▪ No change in dietary pattern in the last five years ▪ No pregnancy or breastfeeding ▪ No history of hormonal and gynecological diseases ▪ No chronic use of methotrexate, cyclosporine, metformin, aspirin, anticonvulsant and contraceptive drugs	▪ Age ▪ Place of residence	Age, marital status, physical activity, number of pregnancies, abortion number, menopause status, age at menopause, dietary pattern
Ghiasvand 2012 [20]	Shiraz	September 2005 to December 2008	986 (493, 493)	Case: 58.2 ± 7.2 Control: 58.0 ± 7.4	Motahari Breast Clinic	Hospital-based	Histopathological confirmation of new breast cancer Aged above 50 years	▪ Healthy female visitors accompanying patients and patients referred to the Faghihi hospital for general surgery, urology, and cardiovascular diseases without any history of breast cancer ▪ No history of hormonal and gynecological diseases	▪ Age (± 5) ▪ Province of residence	Age, education, occupation, BMI, age at menarche, age at first pregnancy, parity, breastfeeding history, breastfeeding duration, OCP use, duration of OCP use, family history of breast cancer
Arbabi Bidgoli 2011 [60]	Tehran	June 2009 to June 2010	150 (50, 100)	Case: 36.45 ± 7.02 Control: 34.2 ± 5.7	Emdad Shahid Beheshti University hospital	Unknown	▪ Histopathological confirmation of new breast cancer ▪ ▪ No menopausal evidence ▪ No pregnancy or breastfeeding	▪ Normal women ▪ No menopausal evidence ▪ No pregnancy or breastfeeding	Age (± 5)	Age, marital status, age at marriage, BMI, weight gain after 18, age at first delivery, age at menarche, irregular menstruation, age at first pregnancy, abortion history, infertility, breastfeeding history, breastfeeding duration, OCP use, duration of OCP use, age at first OCP use, family history of breast cancer, family history of cancer, ovarian cyst
Tehranian 2010 [23]	Tehran	–	624 (312, 312)	Case: 32.2 ± 4.28 Control: 32.9 ± 4.3	Imam Khomeini Hospital	Hospital-based	▪ Histopathological confirmation of new breast cancer ▪ Aged below 40 years	▪Women referred to the same hospital due to non-neoplastic and non-hormonal disease ▪ No diagnosis of cancer or other chronic diseases ▪ Aged below 40 years	▪ Age ▪ ethnicity	Age, marital status, parity, age at first delivery, age at menarche, breastfeeding history, breastfeeding duration, family history of breast cancer, OCP use, abortion history
Ghosn 2020 [61]	Isfahan	–	1050 (350, 700)	Case: 65 ± 11 Control: 61 ± 10	–	population-based	▪ Patients were recruited from those referred to hospitals or private clinics who were undergoing surgical resection of breast cancer, chemotherapy, radiotherapy, or all of the ▪ Histopathological confirmation of new breast cancer ▪ Aged below 40 years ▪ Without any history of any type of neoplastic lesion or cysts ▪ No history of hormone replacement therapy ▪ Without any kind of special diet	▪ Healthy women referring to primary health care centers for their annual personal checkups or attending to receive required information about their children ▪ No relationship with breast cancer patients or had no family history of breast cancer ▪No history of any malignancy, cysts, and medical disorder ▪ No history of hormone replacement therapy Without any type of special diet	▪ Age ▪ ES	Age, education, marital status, place of residence, BMI, physical activity, smoking, supplement intake, dietary pattern, breastfeeding history, menopause status, family history of breast cancer
Saremi 2019 [62] [In Persian]	Arak	March 2017 to May 2018	296 (150, 146)	Case: 46.81 ± 6.96 Control: 44.14 ± 6.52	Ayatollah Khonsari Hospital	Population-based	▪ Breast cancer patients referred to chemotherapy, radiotherapy, or oncology departments ▪ Histopathological confirmation of new breast cancer ▪ No hysterectomy ▪ Aged 20–55 years ▪ No history of organ transplantation ▪ No history of ovarian surgery ▪ Residing in Arak for at least three years ▪ Do not interrupt the menstrual cycle	▪ Neighbors with no history of breast cancer ▪ No pregnancy ▪ No history of chronic disease (diabetes, cardiovascular disease) ▪ No hysterectomy	Age	Age, BMI, physical activity, marital status
Lotfi 2008 [63]	Yazd	February 2006 to December 2006	160 (80, 80)	Case: 48.9 ± 9.7 Control: 49.1 ± 9.8	–	population-based	▪ Breast cancer patients from different clinical centers are involved in diagnostic and treatment of disease ▪ Histopathological confirmation of new breast cancer ▪ Aged 30–75 years ▪ Residing in the urban area of Yazd city	Healthy women from the community of Yazd city according to the place of residence of cases	Age (± 2)	Age, education, occupation, marital status, age of first marriage, menopause status, OCP use, age at menarche, number of live birth, breastfeeding duration, BMI, history of radiation exposure, family history of breast cancer

*OCP *= oral contraceptive, *SES* = social economic status, *BMI* = body mass index, *HRT* = hormone replacement therapy, *WHR* = waist-to-hip ratio

^a^All the mentioned risk factors in each article have not been considered in the analyses for two reasons: 1 Lack of similar reference group, 2- existence of risk factors in only one article

### Risk of bias assessment

The risk of bias assessment was scored by the Newcastle–Ottawa Scale (NOS) from 0 to 9 stars [12]. It was divided into three groups of 0–3 (fair), 4–6 (moderate), and 7–9 (good).

### Statistical analysis

Pooled measures were calculated based on a random-effect model [13]. The heterogeneity was assessed by statistical testing: Cochran's Q (*χ*^2^) test and *I*^2^. Quantitative assessment of heterogeneity was performed on the I^2^ and Higgins classifications. The heterogeneity < 50% was defined as low, between 50 and 74% as moderate, and ≥ 75% as high [14, 15]. The possibility of publication bias was explored by the Egger [16] and Begg [17] tests. If there was publication bias, the Trim and Fill method was employed [18]. A sensitivity analysis was conducted to evaluate the effect of each included study on the final measurement. A significant level was considered for heterogeneity (*χ*2) 0.1 and publication bias and pooled effects 0.05. The data were analyzed using Stata version 14.2 (StataCorp., College Station, TX, USA).

## Results

### Study selection

**Fig. 1:**
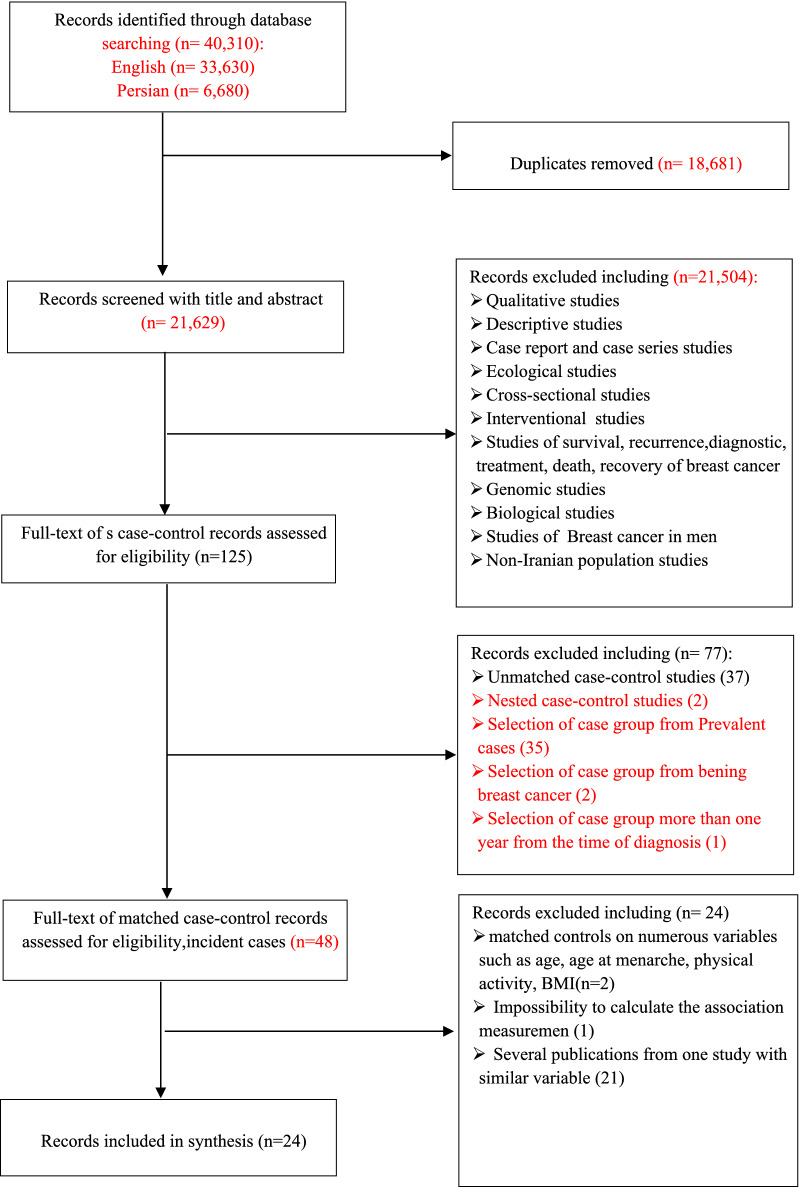
Literature search flowchart for selection of primary studies

A total of 40,310 studies were retrieved, of which 33,630 were in English and 6680 were in Persian. Also, 18,681 articles were excluded due to duplication. After reading the title and abstract of 21,629 articles, the full text of 125 case–control studies was reviewed. In evaluating the full texts, there were two nested case–control studies which we assumed to be valuable for including in the study. But they had studied various and uncommon risk factors different from other included articles, so it was not possible to pool them in our analysis. In this step, 101 records were excluded because of unmatched case–control and unrelated nested case–control studies, the selection of cases and controls not meeting our inclusion criteria, the impossibility of calculating the association measurement, and multiple publications from one dataset. Finally, 24 matched case–control records (22 studies) were investigated in the meta-analysis. Figure 1 shows the steps for screening and selecting articles.

### Study characteristics

A total number of 24 matched case–control records, involving 22 studies were included in this meta-analysis. The included records were conducted in nine cities as Tehran (10), Shiraz (3), Tabriz (2), Tehran/Tabriz (1), Kermanshah (1), Sabzevar (1), Babol (3), Isfahan (1), Arak (1), and Yazd (1) with the published date between 2008 and 2020. In the included studies, 12,460 participants (5675 newly diagnosed cases of breast cancer and 6785 control) were assessed, with the mean age ranging from 32.2 to 65 years in cases and 32.9–61 years in control groups. Control groups in 17 research had been selected from hospitals and clinics, and three research were from the general population. Also, in the four records, the place of control group selection was selected were not clear. Table 2 presents the information on the selected records in detail.

### Risk of bias assessment

All the records were evaluated as moderate and high quality, with scores ranging from 4 to 8. Overall, the risk of bias score of 19 records was moderate and others were good (Table 3).

**Table 3:** Risk of bias assessment

First author, year	Item and score								Total score
	Is the case definition adequate? (1)	Represent activeness of the cases (1)	Selection of controls (1)	Definition of controls (1)	Comparability of cases and controls based on the design or analysis (2)	Ascertainment of exposure (1)	The same method of ascertainment for cases and controls (1)	Non-response rate (1)	
Maleki, 2020 [24]	1	1	0	1	2	1	1	0	7
Safabakhsh, 2020 [48]	1	1	0	1	2	1	1	0	7
Sasanfar, 2019 [26]	1	1	0	0	2	0	1	0	5
Vahid, 2018 [19]	1	1	0	0	2	0	1	0	5
Heidari, 2018 [49]	1	0	0	1	2	0	0	0	4
Fararouei, 2018 [22]	1	1	0	1	2	1	1	0	7
Dianatinasab, 2017 [21]	1	1	0	1	2	1	1	1	8
Pourzand, 2016 [50]	1	1	0	1	2	0	1	0	6
Montazeri, 2016 [51]	1	1	0	1	2	0	1	1	7
Salarabadi, 2015 [52]	1	1	0	0	1	0	1	0	4
Arbabi, 2014 [53]	1	1	0	0	1	0	1	0	4
Hosseinzadeh,2014 [54]	1	1	0	1	2	0	1	0	6
Sheikhi, 2014 [25]	1	1	0	0	2	1	1	0	6
Bahadoran, 2013 [55]	1	1	0	1	2	0	1	0	6
Hajian, 2013 [56]	1	1	0	1	2	0	1	0	6
Hajian, 2012 [57]	1	1	0	1	2	0	1	0	6
Hajian, 2011 [58]	1	1	0	1	2	0	1	0	6
Rezaeiian, 2012 [59]	1	1	0	1	2	0	0	0	5
Ghiasvand, 2012 [20]	1	1	0	1	2	0	1	0	6
Arbabi Bidgoli, 2011 [60]	1	0	0	1	1	0	1	0	4
Tehranian, 2010 [23]	1	1	0	1	2	0	0	0	5
Ghosn, 2020 [61]	1	0	1	1	2	0	0	0	5
Saremi, 2019 [62]	1	0	1	1	1	0	1	0	5
Lotfi, 2008 [63]	1	1	1	0	2	0	1	0	6

### Modifiable risk factors

#### Occupation

**Fig. 2:**
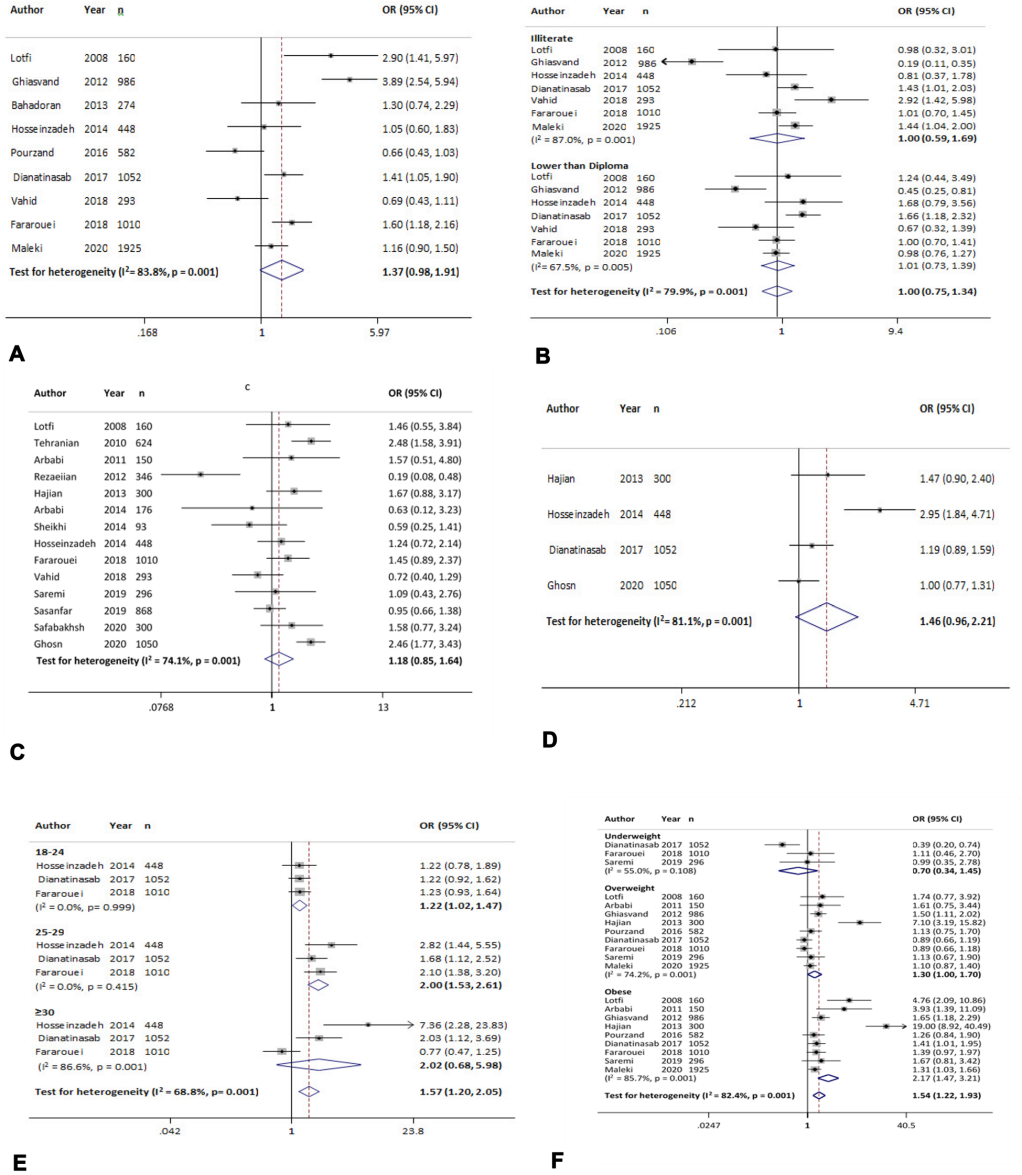
The association between breast cancer and different risk factors. **A**: Occupation (employee vs housewife); **B**: education (lower than university vs university); **C**: marital status (single, divorced, widow vs married); **D**: place of residence (rural vs urban); **E**: age at marriage (≥ 18 vs < 18); **F**: BMI (BMI level vs normal range)

According to nine studies, the overall effect measure showed that employee versus housewife was associated with increased odds of breast cancer by %37 [OR = 1.37 (95% CI 0.98 to 1.91)]; however, this association was not statistically significant (Fig. 2A). The results of the sensitivity analysis showed that excluding each study would change the overall estimate between 1.18 and 1.49 (Table 4).

**Table 4:** Odds ratio, publication bias, heterogenicity, and sensitivity analysis of breast cancer risk factors

Variables	Study (*n*)	*I*^2^ (%)	*χ*^2^	OR (95% CI)	Begg test (*p*_value)	Egger test (*p*_value)	Sensitivity analysis (range of OR)
Modifiable risk factors							
Occupation (ref = housewife)							
Employee	9	83.8	0.001	1.37 (0.98,1.91)	0.53	0.87	1.18–1.49
Education (ref = university)							
Illiterate	7	87	0.001	1.00 (0.59–1.69)	0.45	0.58	0.84–1.32
Lower than diploma	7	67.5	0.005	1.01 (0.73–1.39)	0.88	0.70	0.90–1.14
Marital status (ref = married)							
Single, divorced, widow	14	74.1	0.001	1.18 (0.85–1.64)	0.25	0.09	1.09–1.34
Place of residence (ref = rural)							
Urban	4	81.1	0.001	1.46 (0.96–2.21)	0.17	0.17	1.13–1.69
Age of marriage(ref = < 18)							
18–24	3	0	0.99	**1.22 (1.02–1.47)**	0.60	0.57	1.22–1.23
25–29	3	0	0.41	**2.00 (1.53–2.61)**	0.11	0.34	1.87–2.28
> 29	3	86.6	0.001	2.02 (0.68–5.98)	0.11	0.26	1.23–3.48
BMI (ref = normal)							
Underweight (< 18.5)	3	55	0.10	0.70 (0.34–1.45)	0.60	0.26	0.56–1.05
Overweight (25–29.9)	9	74.2	0.001	1.30 (1.00–1.70)	0.02	0.07	1.11–1.39
Obese (≥ 30)	9	85.7	0.001	**2.17 (1.47–3.21)**	0.06	0.02	1.56–2.37
Physical activity (ref = regular, active)							
moderate, occasionally	6	74	0.002	1.37 (0.82, 2.30)	0.85	0.89	1.12–1.51
Never, seldom	6	86	0.001	1.54 (0.93, 2.54)	0.34	0.51	1.31–2.02
Smoking (ref = no)							
Yes	9	18.9	0.27	**1.83 (1.41–2.38)**	0.53	0.34	1.75- 1.88
Second-hand smoking (ref = no)							
Yes	3	0	0.88	**1.86(1.58–2.19)**	0.60	0.29	1.83–1.90
Alcohol use (ref = no)							
Yes	2	37.6	0.20	0.59 (0.15–2.29)	0.31	–	0.40–2.01
supplement intake (ref = no)							
Yes	5	67.8	0.01	0.61 (0.35–1.07)	0.05	0.04	0.46–0.73
Parity (ref = nulliparous)							
Yes	5	33.2	0.20	0.94 (0.71, 1.24)	0.62	0.94	0.88–1.06
Abortion history (ref = no)							
Yes	5	66.3	0.01	**1.44 (1.02–2.05)**	0.62	0.32	1.21–1.63
OCP use (ref = no)							
Yes	13	74.1	0.001	**1.35(1.11–1.63)**	0.80	0.70	1.26–1.41
HRT (ref = no)							
Yes	5	58.7	0.04	1.03 (0.50–2.14)	1	0.24	0.83–1.31
Breastfeeding history (ref = no)							
Yes	8	0	0.51	0.92 (0.79–1.08)	0.08	0.02	0.88-.0.93
Breastfeeding duration (ref = ≥ 24 months)							
< 24 months	3	79.9	0.007	1.47 (0.74, 2.92)	0.11	0.19	1.09–2.14
Non-modifiable risk factors							
Age of menarche (ref = ≥ 14)							
12–13	4	25.4	0.25	**1.67 (1.31, 2.13)**	0.17	0.45	1.48–1.74
< 12	4	92.7	0.001	2.72 (0.93, 7.99)	0.49	0.34	1.71–4.33
Age of menopause (ref = ≥ 49)							
< 49	3	86.3	0.001	2.03 (0.77–5.34)	0.11	0.02	1.27–3.29
Menopause status (ref = no)							
Yes	12	77.3	0.001	1.18 (0.90–1.55)	0.10	0.30	1.10–1.30
Family history of breast cancer (ref = no)							
Yes	11	73	0.001	**2.47 (1.83–3.33)**	0.39	0.24	2.28–2.65
Family history of cancer (ref = no)							
Yes	4	90.4	0.001	2.57 (0.84–7.85)	1	0.54	1.45–3.58
History of radiation exposure (ref = no)							
Yes	2	0	0.99	**3.48 (2.17–5.59)**	0.31	–	3.46–3.49

Statistical significant OR with P-value less than 0.05 are highlighted in bold

#### Education

In seven evaluated studies, there was no association between illiteracy and the odds of breast cancer [OR = 1.00 (95% CI 0.59 to 1.69)]. The overall estimate changed to 0.84 and 1.32 excluding the studies of Vahid [19] and Ghiasvand [20], respectively. Also, in included articles, no significant association was found between lower than diploma education and the odds of breast cancer [OR = 1.01 (95% CI, 0.73 to 1.39)]. The overall estimate changed to 0.90 and 1.14 excluding the studies of Dianatinasab [21] and Ghiasvand [20], respectively (Fig. 2B, Table 4).

#### Marital status

The overall effect measure of 14 studies showed that single, divorced, and widow versus married was associated with increased odds of breast cancer by %18 [OR = 1.18 (95% CI 0.85 to 1.64)]; however, this association was not statistically significant (Fig. 2C). In sensitivity analysis, the overall estimation changed between 1.09 and 1.34 by excluding each study. Egger test revealed publication bias (*p* = 0.09) (Table 4), but Trim and Fill analysis estimated no censored studies, and OR did not change.

#### Place of residence

The association between residential place and the odds of breast cancer was assessed in 4 studies. No significant association was found between living in urban and the odds of breast cancer [OR = 1.46 (95% CI 0.96 to 2.21)] (Fig. 2D). Sensitivity analysis showed that the overall estimation changed between 1.13 and 1.69 by excluding each study (Table 4).

#### Age at marriage

According to three studies, the age at marriage of 18–24 vs < 18 years was associated with increased odds of breast cancer by %22 [OR = 1.22 (95% CI, 1.02 to 1.47)]. Also, the age group of 25–29 vs < 18 years was significantly associated with odds of two times for developing breast cancer [OR = 2 (95% CI 1.53 to 2.61). This effect was greater in the age group of ≥ 30 vs < 18 years [OR = 2.02 (95% CI, 0.68 to 5.98)] (Fig. 2E, Table 4).

#### Body mass index (BMI)

The overall effect measure in three studies showed that underweight vs normal weight was associated with decreased odds of breast cancer by 30% [OR = 0.70 (95% CI 0.34 to 1.45)]; however, this association was not statistically significant. According to sensitivity analysis, the overall estimate changed to 0.56 and 1.05 excluding the studies of Fararouei [22] and Dianatinasab [21], respectively. Nine studies showed that overweight vs normal weight was significantly associated with increased odds of breast cancer by 30% [OR = 1.30 (95% CI 1.00 to 1.70)]. Also, obesity vs normal weight was significantly associated with odds of 2.17 times for developing breast cancer [OR = 2.17 (95% CI 1.47 to 3.21)]. The tests revealed publication bias, but Trim and Fill analysis estimated no missing studies, and OR did not change (Fig. 2F, Table 4).

#### Physical activity

**Fig. 3:**
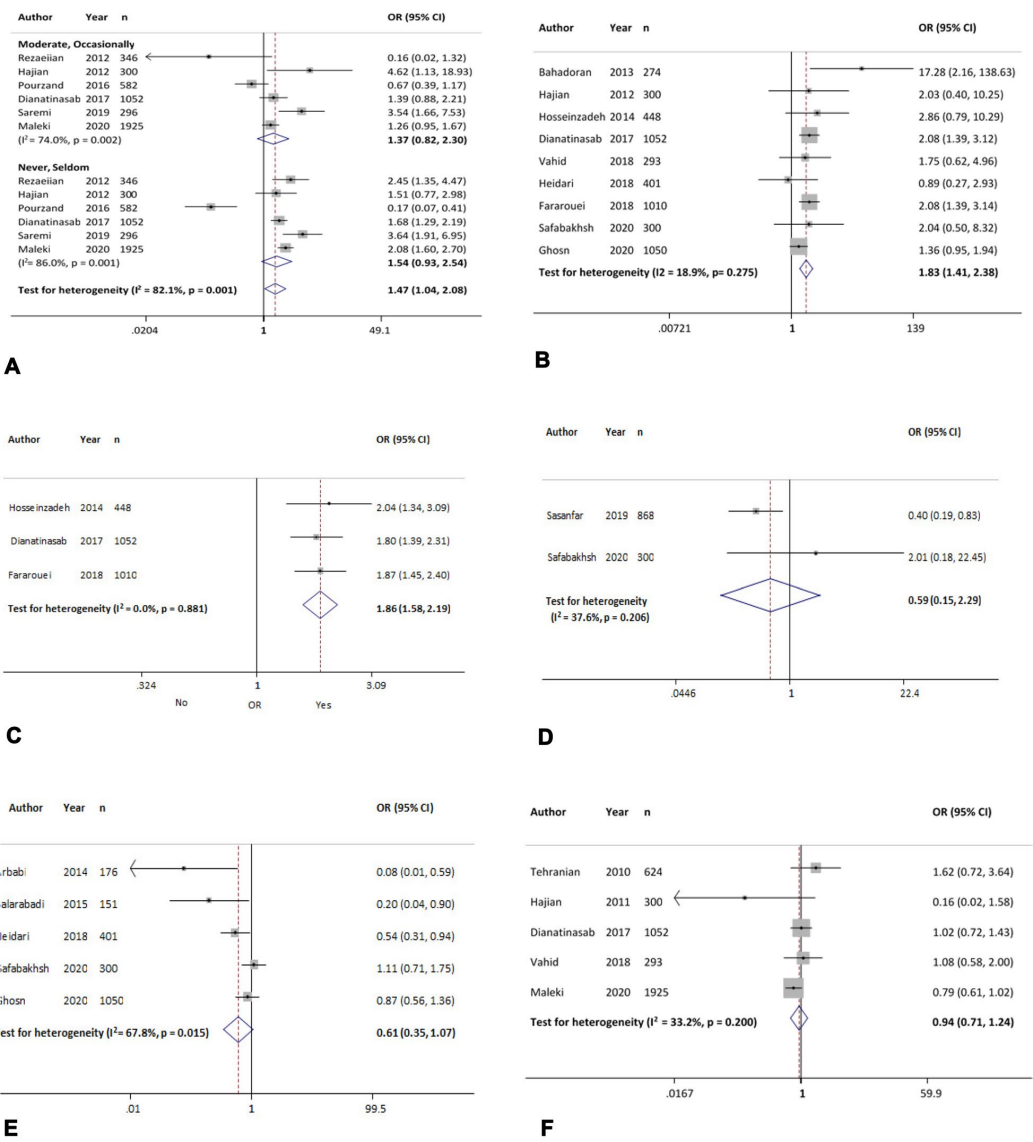
The association between breast cancer and different risk factors. **A**: Physical activity (occasionally and never vs active); **B** smoking (yes vs no); **C**: second-hand smoking (yes vs no); **D**: alcohol use (yes vs no); **E**: supplement intake (yes vs no); **F**: parity (having child vs nulliparous)

The overall effect of six evaluated studies showed that physical activity of occasionally and never versus actively was associated with increased odds of breast cancer by 37% [OR = 1.37 (95% CI 82 to 2.30)] and 54% [OR = 1.54 (95% CI 0.93 to 2.54)], respectively. However, these associations were not significant (Fig. 3A, Table 4).

#### Smoking

Results of nine studies showed that the overall effect of smoking was significantly associated with increased odds of breast cancer up to 83% [OR = 1.83 (95% CI 1.41 to 2.38)] (Fig. 3B). According to sensitivity analysis, the obtained OR for this variable was robust (range of OR changes: between 1.75 and 1.88) (Table 4).

#### Second-hand smoking

The three studies included in this group showed that second-hand smoking was significantly associated with increased odds of breast cancer by 86% [OR = 1.86 (95% CI 1.58 to 2.19)] (Fig. 3C). Sensitivity analysis showed that the obtained OR for this variable had good robustness (range of OR changes: between 1.83 and 1.90) (Table 4).

#### Alcohol use

In two evaluated studies, there was no association between alcohol use and the odds of breast cancer [OR = 0.59 (95% CI 0.15 to 2.29)] (Fig. 3D). According to sensitivity analysis, the overall estimate changed between 0.40 and 2.01 excluding each study (Table 4).

#### Supplement intake

Based on five studies, the overall effect measure showed that supplement intake was associated with decreased odds of breast cancer by 39% [OR = 0.61 (95% CI 0.35 to 1.07)]. However, this association was not significant (Fig. 3E). In sensitivity analysis, overall estimation changed between 0.46 and 0.73 excluding each study. The Begg and Egger test revealed publication bias, but the Trim and Fill analysis estimated no missing studies (Table 4).

#### Parity

According to five evaluated studies, the overall effect measure showed no association between parity and odds of breast cancer [OR = 0.94 (95% CI 0.71 to 1.24)] (Fig. 3F). The sensitivity analysis showed that the overall estimate changes between 0.88 and 1.06 excluding the studies of Tehranian [23] and Maleki [24], respectively (Table 4).

#### Abortion history

**Fig. 4:**
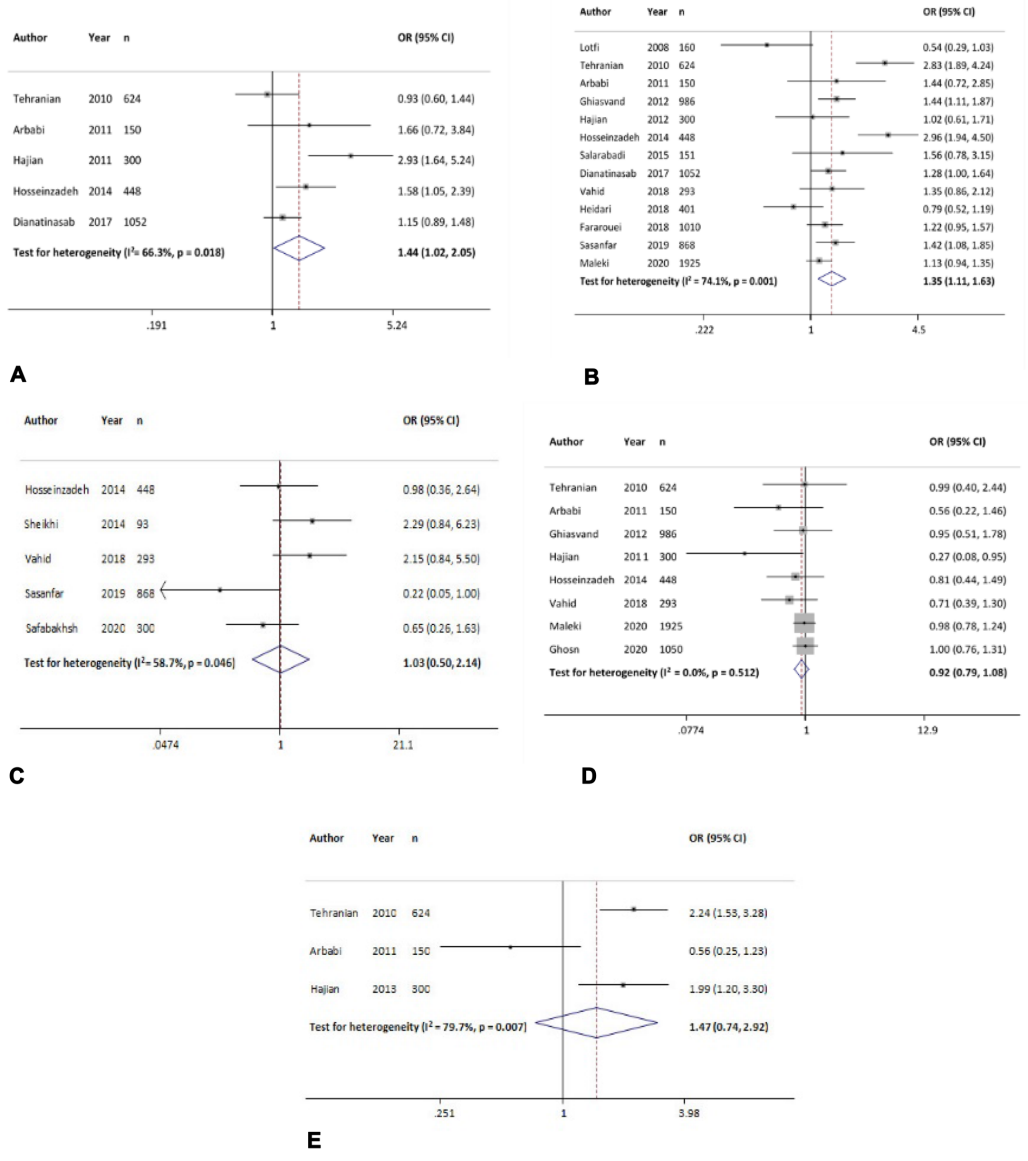
The association between breast cancer and different risk factors. **A**: Abortion history (yes vs no); **B**: OCP use (yes vs no); **C**: HRT (yes vs no); **D**: breastfeeding history (yes vs no); **E**: breastfeeding duration (< 24 months vs ≥ 24 months)

In five studies, the overall effect measure showed that abortion was significantly associated with increased odds of breast cancer by 44% [OR = 1.44 (95% CI 1.02 to 2.05)] (Fig. 4A). According to sensitivity analysis, the overall estimate changed between 1.21 and 1.63 excluding each study (Table 4).

#### Oral contraceptive (OCP) use

According to 13 studies, the overall effect measure indicated that OCP use was significantly associated with increased odds of breast cancer by 35% [OR = 1.35 (95% CI 1.11 to 1.63)] (Fig. 4B). According to sensitivity analysis, the obtained OR for this variable was robust (range of OR changes: between 1.26 and 1.41) (Table 4).

#### Hormone replacement therapy (HRT)

In five evaluated studies, the overall effect measure showed that HRT history was associated with increased odds of breast cancer by 3% [OR = 1.03 (95% CI 0.50 to 2.14)]. However, this association was not significant (Fig. 4C). The sensitivity analysis showed that the overall estimate changes between 0.83 and 1.31 excluding the studies of Sheikhi, Vahid [19, 25], and Sasanfar [26] (Table 4).

#### Breastfeeding history

The overall effect of eight studies indicated that breastfeeding was associated with decreased odds of breast cancer by 8% [OR = 0.92 (95% CI, 0.50 to 2.14)]. However, this association was not significant (Fig. 4D). According to sensitivity analysis, the obtained OR for this variable was robust (range of OR changes: between 0.83 and 0.93). The Begg and Egger test revealed publication bias (Table 4), but missing studies were not found with Trim and Fill analysis and OR did not change.

#### Breastfeeding duration

According to eight studies, the overall effect measure showed that breastfeeding duration < 24 versus ≥ 24 months was associated with increased odds of breast cancer by 47% [OR = 1.47 (95% CI 0.74 to 2.92)]. However, this association was not significant (Fig. 4E). The results of sensitivity analysis showed that the overall estimate changed between 1.09 and 2.14 excluding each study (Table 4).

### Non-modifiable risk factors

#### Age at menarche

**Fig. 5:**
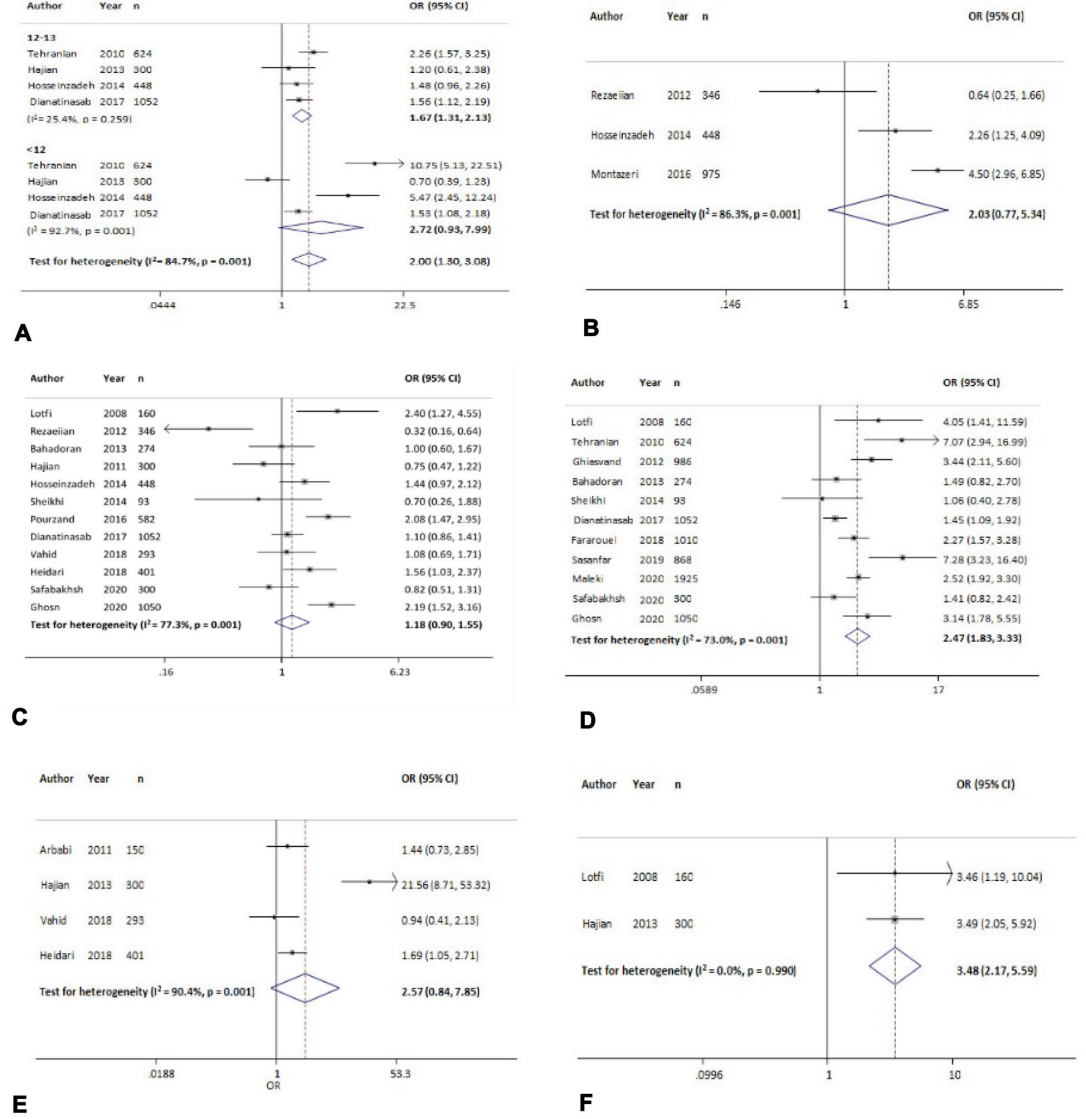
The association between breast cancer and different risk factors. **A**: Age at menarche (< 14 vs ≥ 14); **B**: age at menopause (< 49 vs ≥ 49); **C**: menopausal status (yes vs no); **D**: family history of breast cancer (yes vs no); **E**: family history of cancer (yes vs no); **F**: history of radiation exposure (yes vs no)

Age at menarche was examined in four studies, and the results showed that age at menarche of 12–13 vs ≥ 14 years was significantly associated with increased odds of breast cancer by 67% [OR = 1.67 (95% CI, 1.31 to 2.13)]. Also, the age group < 12 vs ≥ 14 years was associated with odds of 2.72 times for developing breast cancer [OR = 2.72 (95% CI 0.93 to 7.99)] (Fig. 5A, Table 3).

#### Age at menopause

Age at menopause was investigated in three studies and the result showed that age at menopause of < 49 versus ≥ 49 years was associated with odds of 2.03 times for developing breast cancer [OR = 2.03 (95% CI, 0.77 to 5.34)]. However, this association was not significant (Fig. 5B). The Egger test revealed publication bias (*p* = 0.02) (Table 4), but missing studies were not found with Trim and Fill analysis.

#### Menopause status

In 12 evaluated studies, the overall effect measure showed that- menopause was associated with increased odds of breast cancer by 18% [OR = 1.18 (95% CI 0.90 to 1.55)]. However, this association was not significant (Fig. 5C). The results of the sensitivity analysis showed that excluding each study changed the overall estimate between 1.10 and 1.2 (Table 4).

#### Family history of breast cancer

Based on 11 studies, the overall effect measure showed that family history of breast cancer in the first-degree and second-degree relatives significantly was associated with odds of 2.47 times for developing breast cancer [OR = 2.47 (95% CI, 1.83 to 3.33)] (Fig. 5D). Sensitivity analysis showed that the obtained OR for this variable was robust (range of OR changes: between 2.28 and 2.65) (Table 4).

#### Family history of cancer

The overall effect of 11 studies showed that a family history of cancer was associated with odds of 2.57 times for developing breast cancer [OR = 2.57 (95% CI 0.84 to 7.85)]. However, this association was not significant (Fig. 5E). The results of the sensitivity analysis showed that excluding each study changed the overall estimate between 1.45 and 3.58 (Table 4).

#### History of radiation exposure

In two studies, the overall effect measure showed the history of radiation exposure was significantly associated with odds of 3.48 times for developing breast cancer [OR = 3.48 (95% CI 2.17 to 5.59)] (Fig. 5F). The results of the sensitivity analysis showed that excluding each study changed the overall estimate between 3.46 and 3.49 (Table 4).

## Discussion

In this study, extracted breast cancer risk factors were categorized as modifiable and non-modifiable factors. Among the modifiable risk factors, obesity, age at marriage, second-hand smoking, smoking, abortion history, and OCP use were associated with an increased risk of breast cancer. Among the non-modifiable risk factors, history of radiation exposure, family history of breast cancer, and age at menarche increased the risk of developing breast cancer.

### Modifiable risk factors

According to the results of this study, obesity vs normal weight significantly increased the risk of breast cancer. In a meta-analysis study, Liu et al. showed that every 5 units increase in BMI could lead to a 2% increased risk of breast cancer (*P *< 0.001). This dose–response study confirmed a significant linear relationship between BMI and breast cancer risk. In the analysis of premenopausal and postmenopausal subgroups, BMI in the premenopausal group played a protective role in developing breast cancer (*P* < 0.001, 95% CI 0.0–96.99, SRR^1^: 0.98) while it was recognized as a significant risk factor in postmenopausal women (*P* = 0.001; 95% CI 1.1–02.07, SRR = 1.04) [27]. The relationship between increased BMI and the risk of breast cancer has been confirmed in most available sources [28, 29], and certain lifestyle modifications in women can be effective in modulating this risk factor.

Given that sexual relations are often formed in the context of marriage in Iranian culture, Iranian studies emphasize the age of marriage as a risk factor for breast cancer. In the present study, the age at marriage of 18–24 years vs < 18 years increased the risk of breast cancer by 22%, which was also observed in the age of 25–29 vs < 18 years and was significant in both groups. Reports from the Statistical Center of Iran show that the mean age at marriage increased from 25.6 to 27.4 years in men and from 22.4 to 23 years in women during 1996–2016 [30]. Other studies have shown a strong association between the age of the first marriage and the risk of breast cancer [31]. This emphasizes the need for a national policy to facilitate marriage.

The results of the present study confirmed that smoking and second-hand smoke were associated with increased odds of breast cancer by 83% and 86%, respectively. In particular, childhood exposure has been associated with an increased risk of premenopausal cancer [28]. Although smoking is less associated with breast cancer than second-hand smoking in this meta-analysis, this association is underestimated due to information bias. Because in Iran, smoking for women is not generally acceptable, so most women hide their smoking status due to this stigma while second-hand smoking is more presented by them. According to the American Cancer Society in 2019, women who smoked for more than 10 years before their first delivery were 18% more likely to develop breast cancer than non-smokers [28]. Also, a 2013 meta-analysis of 73,000 women showed that smoking before the first delivery significantly increased the risk of breast cancer (hazard ratio = 1.21, 95% CI = 1.14 to 1.28) [32]. Current smoking and past smoking increased the risk of breast cancer by 1.12 and 1.09, respectively [32]. According to US general surgeons’ consensus in 2004, the available evidence was insufficient to establish a causal effect between smoking and breast cancer [33]. Also, in 2009, the International Agency for Research on Cancer stated that there is insufficient evidence that cigarettes are carcinogenic [34]. Despite the significant relationship between smoking and increased risk of breast cancer in the present study, it is necessary to consider limitations such as the timing of smoking and the type of use (continuous or non-continuous). Given lifestyle changes in Iran, and various confounding factors, conducting more comprehensive research can be effective in health policymaking and control of non-communicable diseases.

The results of 5 studies showed that having a history of abortion increased the risk of breast cancer by 44%. A similar meta-analysis of 403,000 women showed an increased risk of developing breast cancer in women with ≥ 3 abortions (OR = 2.39; 95% CI: 1.78–3.21) [35]. Despite the limited number of studies in Iran, the odds ratio is almost equal compared to other studies. Since estrogen as a breast cancer risk factor increases in the first half of pregnancy, abortion exposes undifferentiated breast cells to high concentrations of estrogen during this period [36]. Attention to this pathophysiology emphasizes the importance of preventive measures and opportunistic screening for breast cancer in these individuals.

Thirteen examined studies have shown that the risk of developing breast cancer is significantly increased by 35% by taking oral contraceptive pills. The American Cancer Society states that the recent use of birth control pills is associated with about 20% higher risk of breast cancer, especially before the first pregnancy [28]. OCPs are prescribed as a method of contraception and as a method of treating hormonal disorders. On the other hand, there is no integrated database in Iran that records the duration, amount, and continuity of using these pills. Given the relatively proven role of hormonal compounds in the development of breast cancer, access to the above information can lead to a more accurate estimate of the contribution of hormonal pills in the development of breast cancer.

In nine articles, the risk of developing breast cancer in employed women was 37% higher than that in housewives, although it was not significant at an error level of 5%. The lack of uniform definition and job changes in different periods is an important limitation in examining the causal relationship between occupation and breast cancer. In one meta-analysis, increasing the number of years of jobs with night shift work increased the risk of developing breast cancer by about 1.1 times [37]. The lack of welfare standards in similar occupations is a confounding factor that complicates the study of this relationship. Therefore, multidisciplinary studies that can show the role of occupational factors on breast cancer appear necessary.

In this study, urbanization was associated with an increased risk of developing breast cancer (OR = 1.46; 95% CI 0.96–2.21). In a meta-analysis of 31 studies, Akinyemiju et al. showed a positive relationship between an increased risk of breast cancer and urbanization compared to rural life (Relative Risk = 1.09; 95% CI 1.1–1.19) [38]. The design of population cohort studies in several provinces with appropriate national distribution provides good evidence in this regard.

Physical activity plays a protective role in developing cancer with some hormonal regulation mechanisms such as lowering insulin levels. It is also effective in weight loss, which is indirectly associated with the reduction of breast cancer incidence by lowering insulin levels [39, 40]. Data from 6 studies showed that moderate/occasional physical activity increased the risk of breast cancer by 37% compared to regular exercise. A meta-analysis study of 139 articles, confirms the protective role of physical activity against breast cancer and that this effect size was similar in premenopausal and postmenopausal women [29]. Promoting a healthy lifestyle that includes regular physical activity and a proper diet should be considered a preventative factor in breast cancer.

Data from 47 epidemiological studies in 30 countries showed a 7% reduction in the relative risk of breast cancer with each delivery after adjusting for breastfeeding [41]. The present study also showed a 6% reduction in the risk of breast cancer by having children, which was not statistically significant. Considering the overall decrease in fertility from 2.07 in 2017 to 1.71 in 2020 in Iran [42], addressing this variable can improve the demographic characteristics of Iran and play an influential role in controlling the incidence of breast cancer.

In the present study, the risk of developing breast cancer with HRT after menopause increased by 3%. Other studies confirm this relationship, too [43]. Since the type of hormone consumed has not been reported separately in the studies in Iran, the analysis of this increased risk is worth considering. Certainly, performing breast cancer screening in HRT users increases the likelihood of being diagnosed with breast cancer. Therefore, it is necessary to examine the possibility of undifferentiated misclassification and overdiagnosis.

According to the present meta-analysis, breastfeeding reduced the risk of breast cancer by up to 8%. Also, breastfeeding for less than 2 years compared to 2 years and more increased the risk of breast cancer by 47%. None of these estimates were significant at an error level of 5%. In examining the relationship between breastfeeding and breast cancer, it is necessary to define this exposure more accurately in terms of duration, continuity of time, and quality of breastfeeding.

### Non-modifiable risk factors

In this study, the earlier age of menarche increased the risk of breast cancer. Yi-Sheng et al. showed that the risk of breast cancer decreased by 5–10% for every 1-year delay in menarche [44]. A meta-analysis of 27 studies on Asian women found that age at menarche of 12 years and lower increased the risk of breast cancer by 1.26 times (95% CI 0.93–7.99) [35].

Results showed that the family history of breast cancer in first-degree and second-degree relatives increased the risk of developing breast cancer by 2.47 times, although this estimate did not consider distinguishing between family relationships and the number of people involved. A review study on 113000 women in the UK showed family history in one first-degree relative increased OR by 1.75 times and in the case of two or three people involved, this ratio increased to 2.5 times [45]. Accordingly, in countries with limited resources where population-based screening is not possible, measures for early detection of breast cancer in high-risk populations (positive family history of breast cancer) are recommended.

In two studies, a history of radiation exposure significantly increased the risk of breast cancer by 3.48 times, but this odds ratio is biased because of considering any kind of radiation exposure such as radiography as a risk factor. There was no clear definition for this variable without determining the dose and time of exposure. The results of a systematic review study also showed that a history of radiation exposure to the chest area linearly increased the risk of breast cancer in young women, with a standardized incidence ratio of 13.3 to 55.5 [46]. Our results were derived from only two studies may affect their generalizability and should be interpreted with caution. It seems more accurate for quantitative studies to introduce the attributable risk of radiation exposure in developing breast cancer are needed.

Menopausal age less than 49 years increased the risk of breast cancer by 2.03 times compared to older ages, which was not statistically significant. This contradictory result, in addition to the limited number of studies, could be due to the induced menopause of young patients. Due to the young population composition of Iran, the age of breast cancer incidence is about a decade lower than that in other countries [47] and in most studies, physiological menopause has not been distinguished from induced menopause, which often occurs at a young age. Perhaps the earlier onset of menopause in the case group than that in the control group led to a misclassification of exposure and bias.

Having a family history of cancer increased the risk of breast cancer by 2.57 times. According to the American Cancer Society, a family history of ovarian, pancreatic, and prostate cancer is associated with an increased risk of breast cancer [28]. However, one of the limitations of the present study was the heterogeneity in recording different types of cancers, the number of people involved, and the family relationships of individuals, which makes it difficult to provide a definitive analysis of the risk of this variable in breast cancer.

Some of the variables mentioned could not be pooled because they were only in one of the articles. These variables included socioeconomic status, infertility treatment, night bra use, hair coloring, past life stress, prenatal age, hysterectomy, cosmetic use, day sleep duration, parity number, diabetes, irregular menstruation, and ovarian cyst that were not significantly associated with breast cancer, while variables such as day bra use, sunlight exposure, stress, high-fat diet, migration, history of > 20 kg weight gain after 18 years old were introduced as risk factors and regular bedtime, quality sleep, diet containing sufficient fruit and vegetables, were as protective factors for breast cancer.

### Strengths

One of the issues which make distinguish this meta-analysis from the previous ones is the clarity of the reference group, use of incident cases of breast cancer, lack of combination of different study designs, and conducting sensitivity analysis. This is the first research that has tried to estimate more accurately the attributable risk factors of breast cancer in Iran by considering the methodological limitations of the published studies.

### Limitation

In this study, there are several limitations. Despite the inclusion of studies with newly diagnosed patients, there is still a recall bias publication bias tests were not significant in a small sample size, so the absence of publication bias is not ruled out. In risk of bias assessment, most records had moderate quality due to not selecting the control group from the community and not mentioning ascertainment of exposure and non-response rate. Due to the small number of studies, it was impossible to do metaregression for finding the cause of heterogeneity. Although there were the same risk factors in many studies, due to the lack of similar reference groups, we cannot use all of these studies. There was no complete geographical distribution of breast cancer risk factors. Due to the absence of cohort studies, it is not possible to conclude with certainty the causal relationship of the obtained risk factors. The obtained odds ratios may be overestimated because of not achieving the conditional logistic regression in the primary studies.

## Conclusion

In general, in the present study, age at marriage of 18–29 years, obesity, smoking, second-hand smoking, abortion history, OCP use, age at menarche of 12–13 years, family history of breast cancer, and history of radiation exposure were introduced as risk factors for breast cancer. Given that most of the above are modifiable risk factors, lifestyle changes can play an influential role in the primordial prevention of breast cancer. In women whose risk factors are non-modifiable (women with younger age at menarche, family history of breast cancer, or history of radiation exposure), screening at short intervals can play an effective role in the secondary prevention of breast cancer.

## Supplementary Information

**Additional file 1: Appendix S1.** The search strategy of breast cancer risk factors in Iran.

## Data Availability

The datasets generated and analyzed during the current study are available through contact with the corresponding authors: haghighat@acecr.ac.ir, sha_haghighat@yahoo.com.

## Footnotes

1 Summary Relative Risk.
