# Supplementary material for: Breast cancer risk factors in Iranian women: a systematic review and meta-analysis of matched case–control studies
Source: Eur J Med Res. 2022 Dec 27;27:311. doi: 10.1186/s40001-022-00952-0 (PMC9793603; doi:10.1186/s40001-022-00952-0)
Supplement: Supplementary file 1 — Additional file 1: Appendix S1. The search strategy of breast cancer risk factors in Iran. [file 40001_2022_952_MOESM1_ESM.docx]

**AAppendix 1:** **The search strategy of breast cancer risk factors in Iran**

**Total search strategy in English Database**

((Breast OR Mammary) **AND** (Cancer OR Cancers OR Tumor OR Tumors OR Tumour OR Tumours OR Malignant OR Malignancy OR Malignancies OR Carcinoma OR Carcinomas OR Neoplas OR Neoplasm OR Neoplasms OR Neoplasias)) **AND** (Age OR Race OR Occupation OR Education OR Marital OR Marriage OR residence OR Income OR "Social economic status" OR "SES" OR "body mass index" OR obesity OR "waist circumstance" OR "Physical activity" OR exercise OR smok* OR tobacco OR cigarette OR alcohol OR drug* OR Hookah OR Supplement* OR vegetable* OR diet* OR fat OR food OR sweets OR bra use OR "Hair coloring" OR Cosmetic OR sleep OR "Night shift" OR Stress OR menarche OR menopause OR pregnancy OR delivery OR full term OR Parity OR nullipar* OR Birth OR Abortion OR contracept* OR OCP OR " oral contraceptive use" OR HRT OR hormone OR Infertility OR Breastfeeding OR lactation OR family OR familial OR Relative* OR "first degree" OR " cancer history" OR Malignant OR genetic OR "Blood group" OR radiation OR "x ray" OR Diabetes OR blood pressure" OR "Cyst ovary" OR Hysterectomy OR Biopsy OR mammography OR density OR pesticide* OR Risk OR Risks OR Factor OR Factors OR probability OR Probabilities OR effect* OR affect* OR impact OR Correlat* OR Associat* OR relation* OR Influenc* OR determinant* OR epidemiolog* OR predict* OR predispose* OR assess* OR Evaluat* OR explor* OR etiolog* OR Protect* OR proxy) **AND** (OR Iran OR "Islamic Republic of Iran" OR "IR Iran" OR "I. R. Iran" OR Persia*) **AND** ("1900/01/01" : "2022/11/13").

**Total search strategy in Persian Database**

«سرطان سینه» OR «سرطان پستان» OR «تومور سینه» OR «تومور پستان»

1. **Search strategy formula in Web of Science Database**

| **Web of Science** | |
| --- | --- |
| **Breast Cancer** | (TS= (Breast OR Mammary) AND TS= (Cancer OR Cancers OR Tumor OR Tumors OR Tumour OR Tumours OR Malignant OR Malignancy OR Malignancies OR Carcinoma OR Carcinomas OR Neoplas OR Neoplasm OR Neoplasms OR Neoplasias)) |
| **Iran** | (ALL=(Iran OR "Islamic Republic of Iran" OR "IR Iran" OR "I. R. Iran" OR Persia*)) |
| **Risk Factors** | (TS=("Risk Factors"[Mesh] OR Risk OR Risks OR Factor OR Factors OR probability OR Probabilities OR effect* OR affect* OR impact OR Correlat* OR Associat* OR relation* OR Influenc* OR determinant* OR epidemiolog* OR predict* OR predispose* OR assess* OR Evaluat* OR explor* OR etiolog* OR incidence OR Protect* OR proxy OR age OR family OR familial OR Relative* OR "first degree" OR biops* OR " cancer history" OR Density OR menarche OR menopaus* OR pregnancy OR parity OR nullipar* OR marital OR Abortion OR contracept* OR hormone OR breastfeeding OR lactation OR diabetes OR Stress OR vegetable* OR diet* OR fat OR food OR sweets OR "physical activity" OR exercise OR "body mass index" OR obesity OR smok* OR tobacco OR cigarette OR alcohol OR drug* OR radiation OR pesticide*OR race OR genetic)) |
| **Date** | (PY=(1900-2022)) |
| **Total** | 8470 |

1. **Search strategy formula in Scopus Database**

| **Scopus** | |
| --- | --- |
| **Breast Cancer** | (TITLE-ABS(Breast OR Mammary)) AND (TITLE-ABS(Cancer OR Cancers OR Tumor OR Tumors OR Tumour OR Tumours OR Malignant OR Malignancy OR Malignancies OR Carcinoma OR Carcinomas OR Neoplas OR Neoplasm OR Neoplasms OR Neoplasias)) |
| **Iran** | ALL(Iran OR "Islamic Republic of Iran" OR "IR Iran" OR "I. R. Iran" OR Persia*) |
| **Risk Factors** | TITLE-ABS(Risk OR Risks OR Factor OR Factors OR probability OR Probabilities OR effect* OR affect* OR impact OR Correlat* OR Associat* OR relation* OR Influenc* OR determinant* OR epidemiolog* OR predict* OR predispose* OR assess* OR Evaluat* OR explor* OR etiolog* OR incidence OR Protect* OR proxy OR age OR family OR familial OR Relative* OR "first degree" OR biops* OR " cancer history" OR Density OR menarche OR menopaus* OR pregnancy OR parity OR nullipar* OR marital OR Abortion OR contracept* OR hormone OR breastfeeding OR lactation OR diabetes OR Stress OR vegetable* OR diet* OR fat OR food OR sweets OR "physical activity" OR exercise OR "body mass index" OR obesity OR smok* OR tobacco OR cigarette OR alcohol OR drug* OR radiation OR pesticide* OR race OR genetic) |
| **Date** | (LOAD-DATE < 20221113) |
| **Total** | 9859 |

1. **Search strategy formula in PubMed Database**

| **Pubmed** | |
| --- | --- |
| **Breast Cancer** | "Breast Neoplasms"[Mesh] OR ((Breast[TIAB] OR Mammary[TIAB]) AND (Cancer[TIAB] OR Cancers[TIAB] OR Tumor[TIAB] OR Tumors[TIAB] OR Tumour[TIAB] OR Tumours[TIAB] OR Malignant[TIAB] OR Malignancy[TIAB] OR Malignancies[TIAB] OR Carcinoma[TIAB] OR Carcinomas[TIAB] OR Neoplas[TIAB] OR Neoplasm[TIAB] OR Neoplasms[TIAB] OR Neoplasias[TIAB])) |
| **Iran** | (Iran[Mesh] OR Iran[All Fields] OR "Islamic Republic of Iran"[All Fields] OR "IR Iran"[All Fields] OR "I. R. Iran"[All Fields] OR Persia*[All Fields]) |
| **Risk Factors** | "Risk Factors"[Mesh] OR Risk[TIAB] OR Risks[TIAB] OR Factor[TIAB] OR Factors[TIAB] OR probability[Mesh] OR probability[TIAB] OR Probabilities[TIAB] OR effect*[TIAB] OR affect*[TIAB] OR impact[TIAB] OR Correlat*[TIAB] OR Associat*[TIAB] OR relation*[TIAB] OR Influenc*[TIAB] OR determinant*[TIAB] OR Epidemiology[Mesh] OR epidemiolog*[TIAB] OR predict*[TIAB] OR predispose*[TIAB]OR assess*[TIAB] OR Evaluat*[TIAB] OR explor*[TIAB] OR Etiology[Mesh] OR etiolog*[TIAB] OR Incidence[Mesh] OR incidence[TIAB] OR Protect*[TIAB] OR proxy[TIAB] OR age[TIAB] OR family[Mesh] OR family[TIAB] OR familial[TIAB] OR Relative*[TIAB] OR "first degree"[TIAB] OR Biopsy[Mesh] OR biops*[TIAB] OR "cancer history"[TIAB] OR Density[TIAB] OR Menarche[Mesh]OR Menopause[Mesh] OR menopaus*[TIAB] OR pregnancy[Mesh] OR parity[Mesh] OR nullipar*[TIAB] OR Marriage[Mesh] OR marital[TIAB] OR Abortion[TIAB] OR Contraceptive Agents[Mesh] OR contracept*[TIAB] OR Hormones[Mesh] OR hormone[TIAB] OR "breast feeding"[Mesh] OR lactation[TIAB] OR "diabetes mellitus" [Mesh] OR Stress[TIAB] OR vegetables[Mesh] OR vegetable*[TIAB] OR diet[Mesh] OR diet*[TIAB] OR fat[TIAB] OR food[TIAB] OR sweets[TIAB] OR "physical activity"[TIAB] OR exercise[Mesh] OR "body mass index"[Mesh] OR obesity[Mesh] OR Smoke[Mesh] OR smok*[TIAB] OR tobacco[Mesh] OR "cigarette smoking"[Mesh] OR alcohols[Mesh] OR drug*[TIAB] OR radiation[Mesh] OR pesticides[Mesh] OR pesticide*[TIAB] OR racial groups[Mesh] OR genetics[Mesh] |
| **Date** | "1900/01/01"[PDAT] : "2022/11/13"[PDAT] |
| **Total** | 5783 |

**D. Search strategy formula in Embase Database**

| **Embase** | |
| --- | --- |
| **Breast Cancer** | (breast:ab,ti OR mammary:ab,ti) AND (cancer:ab,ti OR cancers:ab,ti OR tumor:ab,ti OR tumors:ab,ti OR tumour:ab,ti OR tumours:ab,ti OR malignant:ab,ti OR malignancy:ab,ti OR malignancies:ab,ti OR carcinoma:ab,ti OR carcinomas:ab,ti OR neoplas:ab,ti OR neoplasm:ab,ti OR neoplasms:ab,ti OR neoplasias:ab,ti) |
| **Iran** | (Iran OR "Islamic Republic of Iran" OR "IR Iran" OR "I. R. Iran" OR Persia*) |
| **Risk Factors** | risk:ab,ti OR risks:ab,ti OR factor:ab,ti OR factors:ab,ti OR probability:ab,ti OR probabilities:ab,ti OR effect*:ab,ti OR affect*:ab,ti OR impact:ab,ti OR correlat*:ab,ti OR associat*:ab,ti OR relation*:ab,ti OR influenc*:ab,ti OR determinant*:ab,ti OR epidemiolog*:ab,ti OR predict*:ab,ti OR predispose*:ab,ti OR assess*:ab,ti OR evaluat*:ab,ti OR explor*:ab,ti OR etiolog*:ab,ti OR incidence:ab,ti OR protect*:ab,ti OR proxy:ab,ti OR age:ab,ti OR family:ab,ti OR familial:ab,ti OR relative*:ab,ti OR 'first degree':ab,ti OR biops*:ab,ti OR 'cancer history':ab,ti OR density:ab,ti OR menarche:ab,ti OR menopaus*:ab,ti OR pregnancy:ab,ti OR parity:ab,ti OR nullipar*:ab,ti OR marital:ab,ti OR abortion:ab,ti OR contracept*:ab,ti OR hormone:ab,ti OR breastfeeding:ab,ti OR lactation:ab,ti OR diabetes:ab,ti OR stress:ab,ti OR vegetable*:ab,ti OR diet*:ab,ti OR fat:ab,ti OR food:ab,ti OR sweets:ab,ti OR 'physical activity':ab,ti OR exercise:ab,ti OR 'body mass index':ab,ti OR obesity:ab,ti OR smok*:ab,ti OR tobacco:ab,ti OR cigarette:ab,ti OR alcohol:ab,ti OR drug*:ab,ti OR radiation:ab,ti OR pesticide*:ab,ti OR race:ab,ti OR genetic:ab,ti |
| **Date** | [<1900-2022]/py |
| **Total** | -9518 |
